# Pilus distribution among lineages of group b *streptococcus*: an evolutionary and clinical perspective

**DOI:** 10.1186/1471-2180-14-159

**Published:** 2014-06-19

**Authors:** Amber Cody Springman, David W Lacher, Emily A Waymire, Samantha L Wengert, Pallavi Singh, Ruth N Zadoks, H Dele Davies, Shannon D Manning

**Affiliations:** 1 Department of Microbiology and Molecular Genetics, Michigan State University, East Lansing, Michigan, USA; 2 Center for Food Safety and Applied Nutrition, U.S. Food and Drug Administration, Laurel, MD, USA; 3 Institute for Biodiversity, Animal Health and Comparative Medicine, University of Glasgow, Glasgow, UK; 4 Moredun Research Institute, Penicuik, UK; 5 University of Nebraska Medical School, Omaha, NE, USA

**Keywords:** Streptococcus agalactiae, Pilus, MLST, Molecular epidemiology

## Abstract

**Background:**

Group B *Streptococcus* (GBS) is an opportunistic pathogen in both humans and bovines. Epidemiological and phylogenetic analyses have found strains belonging to certain phylogenetic lineages to be more frequently associated with invasive newborn disease, asymptomatic maternal colonization, and subclinical bovine mastitis. Pilus structures in GBS facilitate colonization and invasion of host tissues and play a role in biofilm formation, though few large-scale studies have estimated the frequency and diversity of the three pilus islands (PIs) across diverse genotypes. Here, we examined the distribution of pilus islands (PI) 1, 2a and 2b among 295 GBS strains representing 73 multilocus sequence types (STs) belonging to eight clonal complexes. PCR-based RFLP was also used to evaluate variation in the genes encoding pilus backbone proteins of PI-2a and PI-2b.

**Results:**

All 295 strains harbored one of the PI-2 variants and most human-derived strains contained PI-1. Bovine-derived strains lacked PI-1 and possessed a unique PI-2b backbone protein allele. Neonatal strains more frequently had PI-1 and a PI-2 variant than maternal colonizing strains, and most CC-17 strains had PI-1 and PI-2b with a distinct backbone protein allele. Furthermore, we present evidence for the frequent gain and loss of genes encoding certain pilus types.

**Conclusions:**

These data suggest that pilus combinations impact host specificity and disease presentation and that diversification often involves the loss or acquisition of PIs. Such findings have implications for the development of GBS vaccines that target the three pilus islands.

## Background

Although group B *Streptococcus* (GBS, *Streptococcus agalactiae*) was originally described as a cause of mastitis in bovines, it has emerged as an important opportunistic pathogen in humans. GBS is typically a commensal in the urogenital and lower gastrointestinal tracts of healthy adults, and pregnant women can transmit the bacterium to their baby during childbirth. Newborns infected with GBS can develop life threatening infections including pneumonia, sepsis, and meningitis. GBS has also been shown to cause disease in the elderly and adults with underlying medical conditions where skin and soft tissue infections, urinary tract infections, and bacteremia can result [1].

Molecular epidemiological studies utilizing multilocus sequence typing (MLST) have shown that the distribution of GBS lineages varies by source. Strains belonging to clonal complex (CC)-17 and CC-19, for example, more frequently caused newborn disease compared to strains of other CCs [2-4], with CC-17 strains causing more cases of meningitis and late-onset disease [2]. By contrast, the frequency of strains belonging to CCs 1, 23 and 19 was higher in asymptomatic pregnant women than newborns [3-5], though strains belonging to all three CCs have been frequently isolated from adult and elderly patients with invasive infections [6]. While a subset of CCs have been isolated from both humans and bovines, strains belonging to CC-61 and CC-67 have been found exclusively in cattle [7-10]. Factors that dictate host specificity are poorly understood although several studies have shown that human- and bovine-derived strains have distinct genetic characteristics [7,8,11-13] that may facilitate adaptation to a particular species. Bovine strain FSL S3-026, for instance, was found to have a high frequency of insertion and strain-specific sequences that differed from eight human-derived genomes [13].

Surface adhesins and pili play important roles in GBS adaptation and host specificity. Three pilus islands, (PI)-1, PI-2a, and PI-2b, which encode distinct pilus structures that mediate interactions with host cells, have been identified [14]. Each PI encodes three structural proteins, a backbone protein (BP), two ancillary proteins (AP) and two pilus-specific class C sortase enzymes [15] that recognize LPXTG amino acid motifs on structural proteins and facilitate covalent attachment of these subunits to each other and the cell wall peptidoglycan [16,17]. Differences between PI-1 and the PI-2 variants have been noted [15]. PI-1 is a 16 kb element that integrates between genes *sag0633* and *sag0652* and is flanked by direct repeats, thereby facilitating horizontal gene transfer. PI-2a and PI-2b, however, integrate into one site between genes *sag1410* and *sag1403* and thus, only one or the other can be present in each strain.

*In vitro* models of GBS infection have shown that the APs initiate adherence to various tissues, whereas the BPs facilitate invasion and paracellular translocation of host cells [18-20]. Furthermore, PI-2a was suggested to be more important for biofilm formation [21,22] and the presence of the PI-2b protein, Spb1/SAN1518, was found to increase intracellular survival in macrophages [23]. *In vivo*, GBS pilus components are highly immunogenic and a pilus-vaccine containing the BP genes of PI-1 and PI-2b and the AP of PI-2a has been shown to elicit opsonophagocytic antibodies that confer protection in mice [24].

Given the role that pili play in GBS colonization and disease progression, the type of pilus likely impacts GBS colonization and invasion of host cells. Few studies, however, have characterized the distribution and genetic diversity of each PI in a large population of phylogenetically distinct GBS strains from various sources. Here, we screened for the presence of PI-1, PI-2a and PI-2b in 295 strains recovered from humans and bovines to examine the distribution of each PI across phylogenetic lineages resolved by MLST and identified associations with clinical phenotypes. Genetic variation was also examined in the PI-2 BPs to correlate alleles with phylogenetic lineages, host, and disease. A comprehensive analysis of PIs across diverse strain populations is important to guide current efforts aimed at developing pilus-based GBS vaccines.

## Results

### Phylogenetic analysis

**Figure 1:**
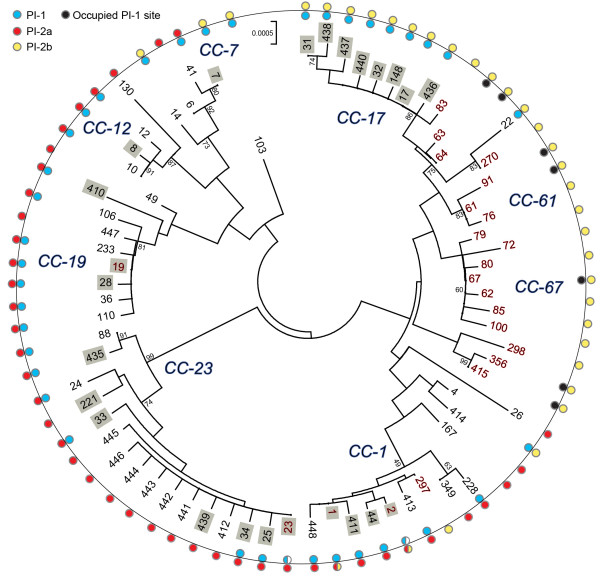
**Evolutionary relationships and pilus island (PI) profiles.** The Neighbor-Joining method was used to infer the evolutionary history among 73 sequence types (STs) representing 3,456 nucleotides, or seven genes. Evolutionary distances were calculated using the p-distance method that represents the number of base differences per site. Numbers at the ends of each branch indicate the STs; grey shading represents human-derived strains from patients with invasive disease while STs shown in red are bovine-derived. Four STs (1, 2, 19, and 23) comprised strains from both humans with and without disease as well as bovines and are indicated in red. The seven clonal complexes (CCs) contained STs that clustered together with significant bootstrap support or that were identified in prior studies. Bootstrap values are indicated at the nodes. Pilus profiles for each ST are shown as colored circles: PI-1 (blue), PI-2a (red), and PI-2b (yellow). Black circles represent those STs containing strains that lacked the PI-1 but possessed an occupied PI-1 integration site.

**Figure 2:**
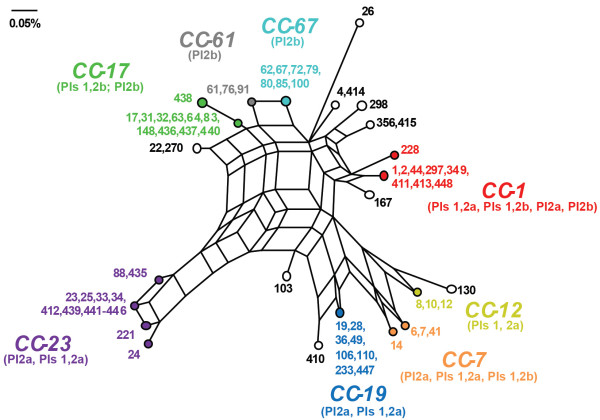
**Recombination among GBS genotypes.** The Neighbor-net analysis highlighted a complex network with evidence of recombination, which is represented as parallelograms, among the 73 multilocus sequence types (STs). Clonal complexes (CCs) are presented in different colors. Closely related STs were collapsed into a single point to improve the clarity of the figure.

Application of MLST to the 295 strains grouped the 73 sequence types (STs) into eight clusters (Figure 1). Although CC-1 had low bootstrap support (49%), we considered it a cluster since our prior study [2] grouped ST-1 with the same STs included in this analysis. The difference in this study was due to the inclusion of the bovine-derived ST-297 strain. The same was true for CC-67, which comprised STs 62, 67, 80, 85, and 100 at 60% bootstrap support. Six singletons (STs 26, 49, 103, 167, 298, and 410) and four smaller clusters were also identified. Neighbor-net analysis provided evidence of recombination among the 73 STs (Figure 2).

Most strains represented CC-19 (*n* = 88; 30%), CC-17 (*n* = 70; 24%), CC-1 (*n* = 36;12%), and CC-23 (*n* = 30; 10%). CC-23 was the most diverse with 16 unique STs, whereas CCs 17, 19 and 1 included nine, seven, and seven STs, respectively. By contrast, CCs 61 and 67 were exclusively comprised of bovine strains, while the remaining bovine strains belonged to three smaller clusters with low bootstrap values or CCs containing mostly human-derived strains. STs 1, 2, 19 and 23 had strains of both human and bovine origin.

### Distribution of PIs across CCs and BP gene variation

**Figure 3:**
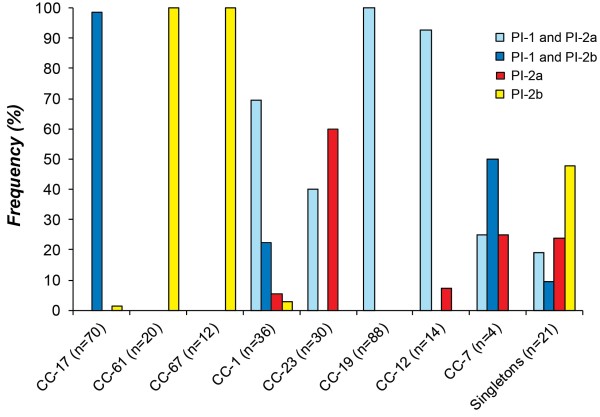
**Frequency of pilus island (PI) types by clonal complexes (CCs).** All 295 stains were screened for the presence of PI-1, PI-2a, and PI-2b using multiplex PCR. The frequency of each PI is illustrated across CCs, which are listed in tree order as determined using the Neighbor-Joining method (Figure 1). Strains representing STs that did not belong to one of the seven CCs were combined into a group of singletons.

Most strains (*n* = 224; 76%) contained PI-1 plus one of the two PI-2 variants, while 71 strains had PI-2a or PI-2b exclusively. PIs were correlated with specific lineages and closely related lineages had similar profiles. While PI-1 had a widespread distribution, the presence of PI-2a and PI-2b was non-random. Within CC’s, little variation was observed in the frequency of PI-2a and PI-2b except in CCs 1 and 7, which had a range of PI profiles. PI-1 frequencies, however, varied within and across CCs, particularly in human strains (Figure 3). Most CC-23 strains (*n* = 18; 60%), for example, lacked PI-1, whereas virtually all CC-19 (*n* = 88; 100%) and CC-17 (*n* = 69; 99%) strains had PI-1 with one PI-2 variant. The only CC-17 strain without PI-1 (ST-83) originated from a bovine. Among strains of the same ST, multiple profiles were observed in two CCs. Within ST-1, all strains had PI-1/PI-2a (*n* = 14) or PI-2b (*n* = 7), while ST-2 strains had three profiles: PI-1/PI-2a (*n* = 6), PI-1/PI-2b (*n* = 1), and PI-2a only (*n* = 1). ST-23 strains had PI-2a with (*n* = 4) and without PI-1 (*n* = 9).

**Figure 4:**
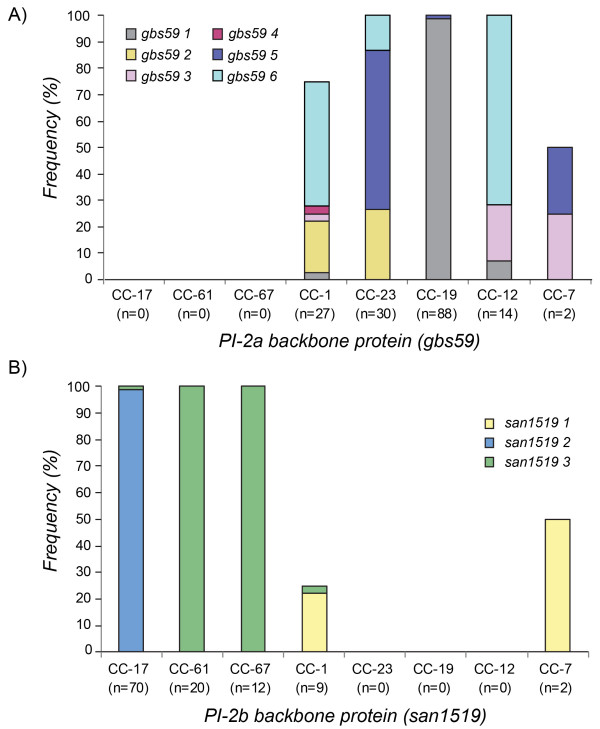
**Frequency of pilus island (PI) backbone protein genes by clonal complex (CC).** The distribution of **A)** six *gbs59* alleles specific for PI-2a is illustrated in 161 group B streptococcal strains and **B)** three *san1519* alleles specific for PI-2b in 113 strains belonging to the seven CCs. In each figure, the CCs are listed in tree order based on the Neighbor-Joining phylogeny (Figure 1). Singletons (n = 21) were excluded from this analysis.

Nine PI-2a/PI-2b BP gene alleles were identified (Additional file 1: Figure S1) and varied across strains (Figure 4). Strains with PI-2a frequently had *gbs59* alleles 1 (*n* = 89; 30%) or 6 (*n* = 32; 11%) while strains with PI-2b had *san1519* alleles 2 (*n* = 69; 23%) or 3 (*n* = 45; 15%). Little variation was observed in *gbs59* among CC-19 strains and in *san1519* among CC-17, -61, and -67 strains. The remaining CCs were more diverse. CC-1 strains, for example, had five of six *gbs59* alleles.

### Epidemiological associations and host specificity

Bovine strains were less variable than human strains with respect to the presence of specific PIs. All bovine strains representing the 18 bovine-specific lineages lacked PI-1, though PI-1 was present in six of the seven bovine strains classified as STs 1, 2, 19, and 23 that contain mostly human-derived strains. Among the 45 PI-1-negative bovine strains, the integration site was occupied by a genetic element other than PI-1 in 18 (40%); the site was intact in the remaining 27. Because a subset of these strains had genomes available, the lack of PI-1 was confirmed in 10 of the 18 strains examined. More specifically, none of the PI-1 genes were detected in any of the strains even though *sal_0710* was detected, however, it was split between two separate contigs. Occupancy was not restricted to specific STs (Figure 1) and different strains representing bovine-specific STs 61, 67, 91, and 415 had both occupied and intact sites. All 26 human strains lacking PI-1, however, possessed an intact integration site. The three bovine strains of STs 23, 83 and 297, which lacked PI-1 and clustered with human strains belonging to CCs 23, 17, and 1, also had an intact integration site.

PI frequencies also varied by strain source. Among the 51 bovine strains, only six (12%) had PI-1 compared to 218 (89%) human strains. Indeed, human versus bovine strains were significantly more likely to have PI-1 as well as PI-2a (Table 1). Only seven (14%) of 51 bovine strains had PI-2a versus 163 (67%) of 244 human strains; six of these seven bovine strains also had PI-1. By contrast, the bovine strains were significantly more likely to have PI-2b than human strains and most (86%) possessed PI-2b exclusively. Among the human strains, differences in PI frequencies were observed by source. Invasive neonatal strains, for instance, were significantly more likely to have PI-1 and one of the two PI-2 variants when compared to the maternal colonizing strains (Table 1). Specifically, 113 (57%) of the 199 strains with two pilus types were recovered from neonates while only 86 (43%) of maternal colonizing strains had both types. Further, the neonatal invasive strains were significantly more likely to have PI-1 with PI-2b than maternal colonizing strains, though the latter had significantly higher frequencies of PI-1 with PI-2a. No difference was observed in the frequency of PI-2a alone across strains.

**Table 1:** PI distributions among strains isolated from humans and bovines as well as neonates with disease (neonatal invasive) and pregnant women without disease (maternal colonizing

	**Human-derived (*n*** **= 244)**		**Bovine-derived (*n*** **= 51)**			
**Pilus island profile**	**n**	**(%)**	**n**	**(%)**		**Fisher’s exact P-value**
PI-1 and PI-2a (*n* = 143)	137	(56%)	6	(12%)		<0.00001
PI-1 and PI-2b (*n* = 81)	81	(33%)	0	(0%)		<0.00001
PI-2a only (*n* = 27)	26	(11%)	1	(2%)		0.06
PI-2b only (*n* = 44)	0	(0%)	44	(86%)		<0.00001
	**Maternal colonizing (*n*** **= 99)**		**Neonatal invasive (*n*** **= 120)**			
**Pilus island profile**	**n**	**(%)**	**n**	**(%)**	**Chi square**	**P-value**
PI-1 and PI-2a (*n* = 143)	66	(53%)	59	(47%)	6.8	0.009
PI-1 and PI-2b (*n* = 81)	20	(27%)	54	(73%)	14.8	0.0001
PI-2a only (*n* = 27)	13	(65%)	7	(35%)	3.5	0.06
PI-2b only (*n* = 44)	0	(0%)	0	(0%)	--	--

Note: The colonizing versus neonatal strain analysis excludes 76 strains that did not fall into either of the two categories.

Percentages were calculated using the column as the denominator for the top half and row for the bottom half and frequencies were compared using the Likelihood Ratio Chi square (χ^2^) and Fisher’s Exact Test.

Stratification by CCs indicated that the 70 CC-17 strains all contained PI-1/PI-2b except for one bovine strain (ST-83) that lacked PI-1. The frequency of strains with PI-1/PI-2b was higher in CC-17 strains relative to all other strains (Fisher’s *p* < 0.0001) even after excluding bovine strains. A similar finding was observed for CC-19 strains, which were more likely to possess PI-1/PI-2a relative to all other strains (Fisher’s *p* < 0.0001) regardless of *cps* (Additional file 1: Table S3). Among the human strains, however, there was no difference in the PI distribution among neonatal and colonizing strains of CC-17 or CC-19 since virtually all strains from each CC had the same profile even after stratifying by *cps*.

Differences in the allele distribution of the PI BP genes were also observed by source. The 44 bovine strains with PI-2b, for instance, had *san1519* allele 3, whereas only one PI-2b-positive human strain harbored this allele. Human strains more frequently had *san1519* alleles 2 (*n* = 69; 85%) and 1 (*n* = 11; 14%). After stratifying *san1519* alleles by source, strains from neonates more frequently had *san1519* allele 2 relative to maternal colonizing strains (Fisher’s *p* < 0.005). No differences were observed in the *gbs59* allele distribution between PI-2a-positive human strains associated with asymptomatic colonization and neonatal disease.

### PI acquisition and loss

**Figure 5:**
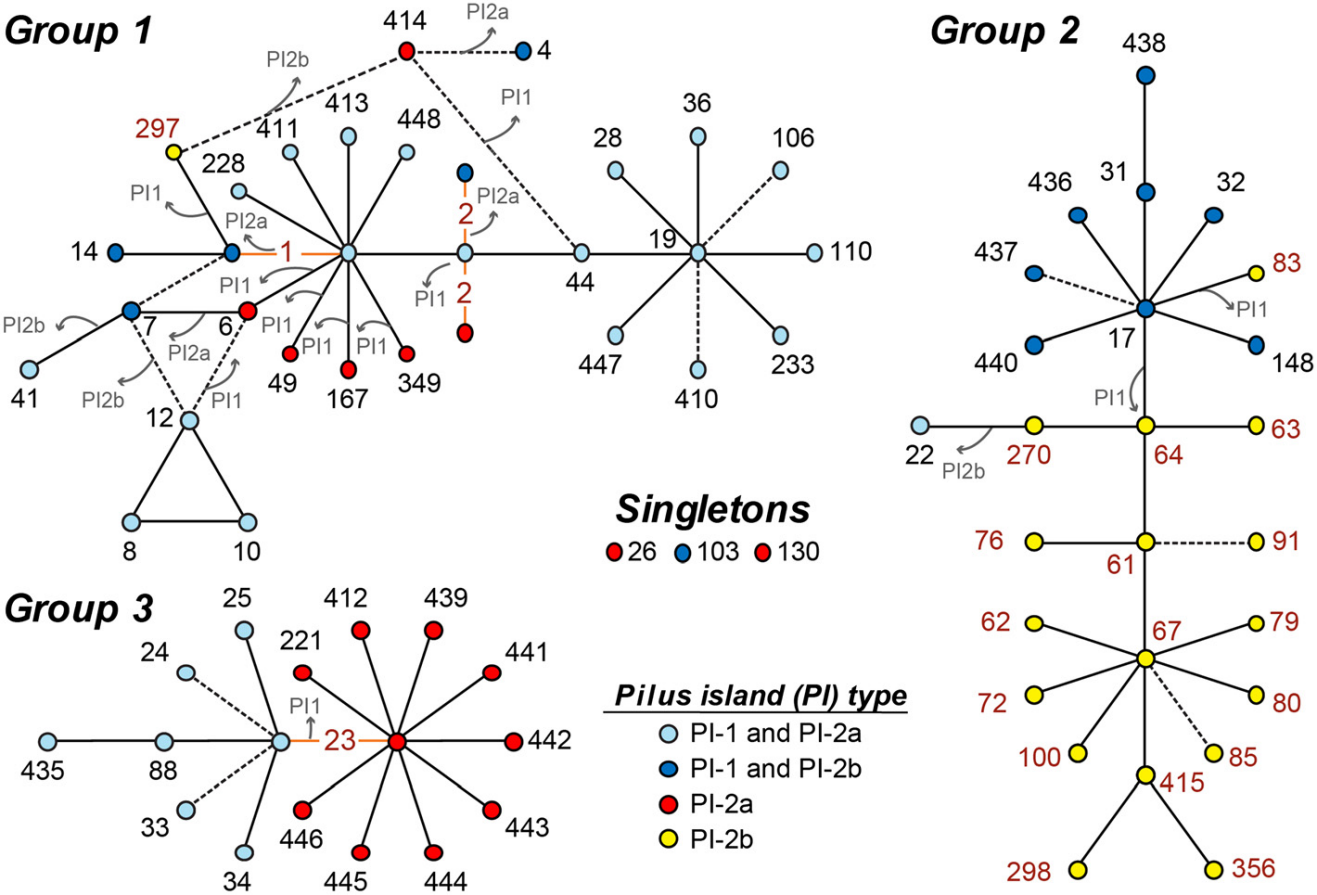
**Gain and loss of pilus islands among GBS sequence types (STs).** eBURST analysis was conducted on the MLST allele profiles for all 295 strains. The founding genotype was assigned to the ST that varies from the largest number of STs at a single locus. STs grouped into three main groups bovine strains indicated by red print. The PI profile distribution is indicated by the color of the circle representing each ST. Double locus variants are connected via dashed lines and STs with multiple pilus profiles are connected with orange lines. In cases where the founding genotype had strains with multiple PI profiles, the ST was listed twice. The predicted founding and co-founding genotypes were used to predict acquisition and loss of PIs, as indicated by the grey arrows.

To model PI-1 acquisition and loss, we mapped the distribution of PI-1 on a phylogenetic tree constructed in eBURST that predicts the ancestral genotypes among the predominant CCs. Three groups and three singletons were identified (Figure 5). PI acquisition and loss occurred frequently in human strains during the diversification of closely related genotypes. PI-1 loss was most common in strains of group 1 since four STs derived from a PI-1 and PI-2a-positive ST-1 strain lost PI-1, while PI-1 was maintained in those genotypes derived from ST-19. Similarly, ST-297, which was isolated from a bovine and is derived from ST-17, lacked PI-1 along with the bovine founder (ST-64) of group 2. Notably, some founding genotypes (e.g., STs 1, 23) were comprised of strains with multiple PI profiles. ST-1 strains, for instance, appear to have diversified into STs with four different PI profiles through the acquisition and loss of PI-1 as well as the exchange of PI-2a for PI-2b. Derivatives of ST-23 strains, however, have maintained one of two profiles following diversification.

## Discussion

As was demonstrated previously, GBS strains from bovines and humans have distinct characteristics that reflect the independent divergence of these two strain populations [7-9,11-13]. The same is true for human-derived strains of different phylogenetic lineages. CC-17 strains, for example, have unique virulence gene alleles [25,26] and PI profiles [27] relative to other CCs, which is likely important for virulence. This analysis of 295 diverse strains from multiple sources in North America provides additional support for these findings, further highlights the complexity of the GBS strain population, and identifies genetic characteristics correlated with strain origin.

The PI distribution observed in this study differs from distributions reported elsewhere in North America, Europe and South Africa [24,27,28]. This difference is largely due to the inclusion of bovine-derived strains in this study and reflects the impact of isolate selection on population level analyses. Most bovine strains had PI-2b exclusively, a profile that was also observed in bovine strains from other geographic locations [9,10] but only in a few human-derived strains [24,27,28]. The difference in PI frequencies between bovine and human strains suggests that pilus types contribute to host specificity. Indeed, most (88%) bovine strains lacked PI-1 unlike the human strains, which more frequently had PI-1 in combination with one of the PI-2 variants. Since 40% of the 45 bovine strains lacking PI-1 had an occupied integration site, it is likely that PI-1 confers an advantage in the human host and is not necessary for colonization in bovines. Interestingly, a PI-1 deletion mutant was found to reduce internalization by human-derived monocytes despite having no effect on attachment to A549 lung epithelial cells, VK2 vaginal cells, or ME180 cervical cells in a prior study [29]. It is therefore possible that PI-1 serves primarily to protect against human-derived phagocytic cells while other adherence factors are more important for GBS colonization of the genitourinary tract. Within bovine strains, PI-1 may represent a metabolic burden to the bacterium and be more susceptible to excision or may lack an accessible integration site that prevents PI-1 incorporation into the genome. BLAST results on the consensus sequences from the occupied integration site in two of the PI-1-negative bovine genomes (ANPW00000000 and ANPS01000000), for example, detected several genes from *Streptococcus dysgalactiae subsp. equisimilis*. A future comprehensive comparative genomics study, however, would be needed to better understand the level of diversity within this integration site in strains with and without PI-1.

A relationship was also observed between PI-1 and phylogenetic lineage. All strains of CCs 17 and 19, the complexes previously linked to neonatal infections [2-4], had PI-1 in combination with another PI-2 variant; most (95%) were serotype III. In human strains of all other lineages, however, many (27%) lacked PI-1 altogether suggesting that it is more important for colonization and disease progression in certain genetic backgrounds. As we have observed the same degree of diversity in many other GBS surface proteins [25,26], it is possible that individual strains utilize different adherence mechanisms to colonize the host. Further stratification by the type of PI-2 variant demonstrated that 98% of neonatal CC-17 strains had PI-1 with PI-2b; none of the strains with this PI profile from other lineages originated from neonates, suggesting that PI-2b may be important for neonatal disease. Interestingly, all 53 *cps*III CC-17 strains contained *san1519* allele 2 encoding the PI-2b BP, the major component of the pilus structure [24], also suggesting a specific role for this allele in neonatal disease. Although the diversity of *san1519* is low, the allelic distribution varied among human and bovine strains with the latter exclusively carrying allele 3. Outside of CC-17, PI-1/2b-positive strains of CC-1 had *san1519* allele 1 and represented rare *cps* types (e.g., IV, VII, and VIII). The extensive genetic diversity seen across CCs reflects the independent divergence of these strain populations and highlights features that may influence host specificity and pathogenic potential. Additional studies are needed, however, to examine whether strains of different lineages and PI profiles have an enhanced ability to colonize and/or invade human epithelial cells. It would also be worthwhile to compare PI distributions among strains associated with uncomplicated infections such as urinary tract infections and wound infections since a prior study identified different STs to be associated with these types of infections [30].

Unlike *san1519*, the PI-2a BP gene, *gbs59*, was diverse in strains of lineages previously associated with maternal colonization (e.g., CC-1 and CC-23). Presumably, diversity within PI-2a enhances versatility and enhances the ability to colonize multiple hosts and niches. Support for this hypothesis comes from the reportedly high frequencies of CCs 1 and 23 in asymptomatic women [5] as well as their isolation from bovines [7,8,31] and other animal species [32,33]. As antigenic variation is important for evasion of host immune responses, the high level of diversity in *gbs59* may be the result of strong selective pressures encountered within different hosts. The presence of identical alleles among unrelated strains (Figure 4) also suggests that *gbs59* is a “hot spot” for recombination, while low sequence variability in *san1519* of PI-2b is evidence of a more constrained evolutionary history.

Because there is a clear correlation between phylogenetic lineage and PI profile, both vertical inheritance and horizontal gene transfer have likely contributed to the PI distribution observed. While the PI-2 variants are likely heritable, the presence of PI-1 is less predictable. For the 30 CC-23 strains examined, PI-1 was present in 12 (40%), which is considerably higher than the frequency detected in CC-23 strains from Spain [27], suggesting that there is considerable geographic variation in PI profiles. Such variation may be due to baseline frequencies of PI-1 in specific populations as it may be more susceptible to horizontal gene transfer, a plausible hypothesis since the island is flanked by direct repeats and contains transposable elements [15]. The absence of PI-1 in CCs unrelated to CC-23 and in specific STs within CC-23 provides additional support for this hypothesis. Following horizontal gene transfer, PI-1 may remain incorporated into the chromosome in some strains, thereby resulting in an increased fitness and colonization potential. Alternatively, it may also be excised from others, which may be due to both host-specific pressures and bacterial stress responses. Indeed, increased horizontal gene transfer and mutation rates have been documented in other pathogens following exposure to certain stressors [34]. Because the GBS PIs are highly immunonogenic [14,24], the loss of PI-1 could also provide a mechanism to evade the host immune responses, a process that could be advantageous to certain genotypes that are more prone to cause invasive disease or after exposure to new niche.

The eBURST analysis demonstrated that the neonatal invasive lineage, ST-17, is related to the ST-67 bovine lineage and suggests that PI-1 was either acquired in the ST-17 strain population or lost in the ST-67 bovine population. Although a close relationship was previously identified between STs 17 and 67 [7], it is important to note that eBURST results are greatly impacted by the number and type of STs included in any given analysis. More recent data of all STs available in the PubMLST database [35] suggest that ST-17 is part of eBURST group 1 with STs 19 and 1, which has subsequently diversified into several host-specific complexes including one containing ST-67 and other bovine-associated STs [33]. Further, it was suggested that the ST-17 subpopulation emerged via a series of evolutionary events including recombination among strains belonging to multiple clonal complexes [9] (Figure 2) as well as the acquisition of mobile genetic elements. This hypothesis is supported by our finding that many of the bovine strains were related to human strains containing PI-1 (e.g., ST 83 and 64, Figure 5) or had a PI-1 integration site occupied by another genetic element (e.g., STs 61, 64 and 67, Figure 5) unlike the human-derived strains. Those bovine strains with an occupied integration site may not be capable of acquiring PI-1, which may limit their ability to be transmitted to and sustained in the human host. Collectively, these data suggest that the human vs. bovine strains more frequently serve as eligible recipients for PI-1, and many human strains have lost PI-1 during diversification.

Although the factors that contributed to the emergence of GBS in human populations are not fully understood, acquisition of PI-1 through horizontal gene transfer may have facilitated this process. PI-1 likely increased the fitness and colonization potential of some strains within the human host, thereby allowing them to establish a niche within a pregnant mother, for instance, and enhancing the likelihood of an opportunistic infection and subsequent transmission to a susceptible neonate. Additional studies, however, are required to test whether strains with different STs and PI profiles vary in their ability to colonize, persist, and invade host tissues relevant to the disease process. In the meantime, enhancing our understanding of PI distribution patterns and genetic diversity in strains from different sources and geographic locations is critical for future efforts aimed at the development of pilus-based GBS vaccines, which were effective in neonatal mice [24,27]. The variable presence of PI-1 among human strains and the possibility of PI-1 loss *in vivo* may limit protection elicited through a vaccine targeting PI-1 alone. Consequently, enhancing our understanding of PI distribution patterns and genetic diversity in strains from different sources and geographic locations is critical for future efforts aimed at the development of pilus-based GBS vaccines, which were effective in neonatal mice [24,27]. The variable presence of PI-1 among human strains and the possibility of PI-1 loss *in vivo* may limit protection elicited through a vaccine targeting PI-1 alone.

## Conclusions

The analysis of 295 isolates from diverse sources demonstrated significant variation in the distribution of PI types across phylogenetic lineages and sources, suggesting that pilus combinations impact host specificity and disease outcomes. Moreover, we observed that diversification of specific GBS lineages within certain populations can involve the loss or acquisition of PIs. The variable presence of specific PIs has considerable implications for the development of GBS vaccines targeting these pili.

## Methods

### Bacterial population

A total of 295 bacterial isolates were included in the study. Most isolates were originally recovered from neonatal blood or cerebral spinal fluid (invasive isolates; *n* = 120) [36] and vaginal/rectal swabs of pregnant women (maternal colonizing isolates; *n* = 89) [37]. Approval to collect specimens was granted by the University of Calgary Ethics Board; informed consent was obtained prior to sample collection. Approval to characterize the de-identified bacterial isolates was provided by both the University of Calgary Ethics Board and Michigan State University Institutional Review Board.

Isolates were characterized by multilocus sequence typing to group isolates in to sequence types (STs) and clonal complexes (CCs). All CC-17 and -19 strains from 98 (out of 192) newborns [2] and 43 (out of 194) pregnant women [5] were included to compare PI frequencies among isolates from the same time and location. Fifty-one isolates from bovines with clinical or subclinical mastitis [7,8] were included to compare PI distributions to human-derived isolates as was a reference set of 80 human-derived GBS strains of varying STs and serotypes [26]. Cultures were grown in Todd-Hewitt broth at 37°C with 5% CO_2_ and capsule (*cps*) types were determined for a subset of strains as described [38].

### Phylogenetic analysis

Seven housekeeping genes commonly used for MLST [3] were sequenced and a Neighbor joining phylogeny [39] with 1,000 bootstrap replications was constructed in MEGA5 [40]. Groups of three or more STs with >80% bootstrap support or that were defined in prior studies were considered to represent the CCs; all were originally uncovered by BURST [3]. Recombination was examined in SplitsTree4 [41], while eBURSTv3 [42] was used to identify ancestral genotypes and map PI acquisition and loss.

### GBS PIs distribution and variation

PCR amplification of genes encoding sortase C [*sag647*, *sag1406* and *san1517*] and *adhP* was performed (Additional file 1: Table S1) and PI frequencies were compared by source and ST. For PI amplification by PCR, 2 mM dNTP was added to 25 mM MgCl_2_, 10 mM primers, 10X buffer II, 1.5 U AmpliTaq Gold (Applied Biosystems), 15 ng/μl DNA and ddH_2_0 in a 25 μl final volume. Thermocycling conditions utilized an initial soak of 94°C for 10 min, followed by 35 cycles of: 92°C for 1 min, 53°C for 1 min, and 72°C for 30 sec; and a final step of 72°C for 5 min. Strains lacking PI-1 were screened using primers targeting *sal_0710*, which represents the integration site from GBS genome strain 515 (NZ_AAJP01000027) as described by Martins et al. [43]. Amplification of a 684 bp fragment indicated an intact integration site and no amplification indicated occupancy by a genetic element other than PI-1 [43]. The latter was confirmed by examining the occupied region in 12 published genomes, which included a subset of the PI-1-negative bovine strains examined as part of this study. The genomes included the following: ANPS01000000, ANPW00000000, ANQA00000000, ANPU00000000, ANPT00000000, ANPX01000000, ANPY00000000, ANQF00000000, ANCM00000000, ANPZ00000000, ANCK00000000, and ANCO00000000. BLAST was used to search for the ten known PI-1 genes, *sag0642*-*sag0651*, within the region along with the PI-1 integration site.

Variation within the BP gene of PI-1 was not examined as only 19 of 9,594 nucleotides varied across the six genome strains; however, *in silico* analysis of a subset of genomes [44-46] was performed to identify restriction enzymes (*Pvu*II and *Ssp*I) capable of differentiating PI-2a and PI-2b BP genes, *gbs59* and *san1519* (Additional file 1: Table S2). For amplification of *gbs59*, PCR was performed in a 25 ul reaction with 10 mM of primers and LA *Taq* (Takara Bio, Inc.) using the following conditions: initial soak at 94°C for 1 min, followed by 30 cycles of 94°C for 30 sec, 54°C for 30 sec, and 68°C for 3 min, and a final soak at 72°C for 10 min. Amplification of *san1519* used the same cycling conditions with a higher annealing temperature (55°C) and shorter extension time (1.5 min). *gbs59* was digested with *Pvu*II (New England BioLabs, Inc.), while *Ssp*I (New England BioLabs, Inc.) was used for *san1519*.

## Competing interests

The authors declare no competing interests.

## Authors’ contributions

ACS, SDM, HDD designed the study; ACS, EAW, SLW, PS performed the work and interpreted molecular and genomic data; ACS, DWL developed molecular assays; ACS, DWL, RNZ, HDD, SDM analyzed epidemiological and evolutionary data and drafted the manuscript. All authors read and approved the final manuscript.

## Supplementary Material

Additional file 1: Table S1 Comparison of pilus island type distributions among strains by group B streptococcal clonal complex (CC) and capsule (*cps*) type. **Table S2.** Pilus island (PI) multiplex PCR with gene targets, primer sequences, and expected size fragments. PCR targeting *sag647* (PI-1), *sag1406* (PI-2a), and *san1517* (PI-2b) was used to determine which PIs were present, while PCR-based restriction fragment length polymorphism (RFLP) analysis was used to amplify the PI-2 variant backbone protein (BP) genes, *gbs59* (PI-2a) and *san1519* (PI-2b). **Table S3.** PCR-based RFLP for backbone protein (BP) genes of pilus island (PI)-2a and PI-2b. Digestion of the PI-2a BP gene, *gbs59*, with *Pvu*II yielded six major alleles, while *Ssp*I digestion of the PI-2b BP gene, *san1519*, yielded three alleles. The representative GenBank reference sequences for each variant are listed along with the average size of the expected fragments based on *in silico* analyses. **Figure S1.** Allelic variation in the backbone protein (BP) genes of the pilus island (PI) 2 variants. A) Neighbor-joining phylogeny of the PI-2a BP gene, *gbs59*, based on an *in silico* analysis of 23 published sequences available in GenBank. Six major alleles were identified with 1,273 differences in 2,163 nucleotides and sorted into two groups: group 1 contains alleles, 1, 2, and 3, and group 2 contains alleles 4, 5, and 6. Bootstrap values based on 1000 replications are indicated at the nodes. B) Neighbor-joining phylogeny of thee alleles of the PI-2b BP gene, *san1519*, based on an *in silico* analysis of three published sequences. *san1519* alleles 1 and 2 differ at 199 of 4,317 nucleotides, whereas alleles 2 and 3 differ at 54 sites. Strain FSL S3-026, indicated in red, represents a bovine strain. Click here for file
