# Supplementary material for: Pilus distribution among lineages of group b streptococcus: an evolutionary and clinical perspective
Source: BMC Microbiol. 2014 Jun 19;14:159. doi: 10.1186/1471-2180-14-159 (PMC4074840; doi:10.1186/1471-2180-14-159)
Supplement: Additional file 1: Table S1 — Comparison of pilus island type distributions among strains by group B streptococcal clonal complex (CC) and capsule (cps) type. Table S2. Pilus island (PI) multiplex PCR with gene targets, primer sequences, and expected size fragments. PCR targeting sag647 (PI-1), sag1406 (PI-2a), and san1517 (PI-2b) was used to determine which PIs were present, while PCR-based restriction fragment length polymorphism (RFLP) analysis was used to amplify the PI-2 variant backbone protein (BP) genes, gbs59 (PI-2a) and san1519 (PI-2b). Table S3. PCR-based RFLP for backbone protein (BP) genes of pilus island (PI)-2a and PI-2b. Digestion of the PI-2a BP gene, gbs59, with PvuII yielded six major alleles, while SspI digestion of the PI-2b BP gene, san1519, yielded three alleles. The representative GenBank reference sequences for each variant are listed along with the average size of the expected fragments based on in silico analyses. Figure S1. Allelic variation in the backbone protein (BP) genes of the pilus island (PI) 2 variants. A) Neighbor-joining phylogeny of the PI-2a BP gene, gbs59, based on an in silico analysis of 23 published sequences available in GenBank. Six major alleles were identified with 1,273 differences in 2,163 nucleotides and sorted into two groups: group 1 contains alleles, 1, 2, and 3, and group 2 contains alleles 4, 5, and 6. Bootstrap values based on 1000 replications are indicated at the nodes. B) Neighbor-joining phylogeny of thee alleles of the PI-2b BP gene, san1519, based on an in silico analysis of three published sequences. san1519 alleles 1 and 2 differ at 199 of 4,317 nucleotides, whereas alleles 2 and 3 differ at 54 sites. Strain FSL S3-026, indicated in red, represents a bovine strain. [file 1471-2180-14-159-S1.pdf]

Supplemental Table 1. Comparison of pilus island type distributions among strains by group B streptococcal clonal complex (CC) and capsule (*cps*) type.

		**Pilus island (PI) profile**							
		**PI-1 and PI-2a (*n*=141)**		**PI-1 and PI-2b (*n*=81)**		**PI-2a only (*n*=27)**		**PI-2b only (*n*=43)**	
**CC**	***cps***	**n**	**(%)**	**n**	**(%)**	**n**	**(%)**	**n**	**(%)**
CC-1 (n=36)	Ia	-	-	-	-	-	-	1	(2.3%)
	II	1	(0.7%)	-	-	1	(3.7%)	-	-
	III	1	(0.7%)	-	-	-	-	-	-
	IV	4	(2.8%)	1	(1.2%)	-	-	-	-
	V	11	(7.8%)	-	-	1	(3.7%)	-	-
	VI	7	(5.0%)			-	-	-	-
	VII	-	-	2		-	-	-	-
	VIII	-	-	5		-	-	-	-
	NT	1	(0.7%)			-	-	-	-
CC-7 (n=4)	Ia	-	-	1	(1.2%)	-	-	-	-
	Ib	-	-	-	-	1	(3.7%)	-	-
	V	1	(0.7%)	-	-	-	-	-	-
	VI	-	-	1	(1.2%)	-	-	-	-
CC-12 (n=14)	Ib	9	(6.4%)	-	-	-	-	-	-
	II	3	(2.1%)	-	-	-	-	-	-
	V	1	(0.7%)	-	-	-	-	-	-
	VIII	-	-	-	-	1	(3.7%)	-	-
CC-17 (n=70)	II	-	-	-	-	-	-	1	(2.3%)
	III	-	-	69	(85.2%)	-	-	-	-
CC-19 (n=88)	Ia	1	(0.7%)	-	-	-	-	-	-
	Ib	1	(0.7%)	-	-	-	-	-	-
	II	12	(8.5%)	-	-	-	-	-	-
	III	72	(51.0%)	-	-	-	-	-	-
	V	2	(1.4%)	-	-	-	-	-	-
CC-23 (n=28)	Ia	6	(4.2%)	-	-	15	(56.0%)	-	-
	II	-	-	-	-	1	(3.7%)	-	-
	III	4	(2.8%)	-	-	1	(3.7%)	-	-
	NT	-	-	-	-	1	(3.7%)	-	-

CC-61 (n=20)	II	-	-	-	-	-	-	6	(14.0%)
	III	-	-	-	-	-	-	10	(23.3%)
	VII	-	-	-	-	-	-	1	(2.3%)
	NT	-	-	-	-	-	-	3	(7.0%)
CC-67 (n=11)	II	-	-	-	-	-	-	11	(25.6)

Note: The 21 singletons were omitted from the analysis as were four strains with missing *cps* data.

Supplemental Table 2. Pilus island (PI) multiplex PCR with gene targets, primer sequences, and expected size fragments. PCR targeting *sag647* (PI-1), *sag1406* (PI-2a), and *san1517* (PI-2b) was used to determine which PIs were present, while PCR-based restriction fragment length polymorphism (RFLP) analysis was used to amplify the PI-2 variant backbone protein (BP) genes, *gbs59* (PI-2a) and *san1519* (PI-2b).

PI	Assay	Gene target	Sequence	Size
1, 2a, 2b	PCR	adhP_F162	ACGCATTTTGGGTCACGA	783 bp
		adhP_R944	GTATCCACAGGCACTTTTTCAAC	
1	PCR	SAG647_F496	CTACCAACGGCCAAGCTATTTACC	394 bp
		SAG647_R889	TAGCCGCTTTTTCATTCTTTCTCC	
2a	PCR	SAG1406_F356	AACTCCCTATATTTGCAGGTTCAA	243 bp
		SAG1406_R598	CGGGTGTAACGACTTTTATCTGAT	
	RFLP	GBS59_Fup	CAACACGCATATTCCACCAAAAG	2.8 kb
		GBS59_Rdn	TCTAACATACGGGCGTACTCTGCT	
2b	PCR	SAN1517_F57	GGGGGTAGGCTTAATGGCTTAT	519 bp
		SAN1517_R575	TCCGGTTTAACTGTTCTGATTTGAT	
	RFLP	SAN1519_F1485	GCCCGCACAAACAACCTACAC	1.7 kb
		SAN1519_R3167	AAATGGGCGTCAATATCAATGGA	

Supplemental Table 3. PCR-based RFLP for backbone protein (BP) genes of pilus island (PI)-2a and PI-2b. Digestion of the PI-2a BP gene, *gbs59*, with *Pvu*II yielded six major alleles, while *Ssp*I digestion of the PI-2b BP gene, *san1519*, yielded three alleles. The representative GenBank reference sequences for each variant are listed along with the average size of the expected fragments based on *in silico* analyses.

PI	Major Allele	Reference Sequence	Fragment sizes					
2a	1	18RS21			39	261	983	1546
2a	1	2603V-R			39	261	983	1546
2a	2	EU929876	33	294	303	513	725	955
2a	3	H36B			336	336	664	1443
2a	4	EU929869					733	2024
2a	5	515					1264	1472
2a	5	EU929888					1264	1508
2a	6	CJB111				609	949	1168
2a	6	NEM316				612	949	1165
2a	6	EU929963				609	949	1393
2b	1	A909		81	251	599	749	
2b	2	COH1	171	251	311	351	599	
2b	3	FSL03-26		171	251	599	662	

Fig. S1. Allelic variation in the backbone protein (BP) genes of the pilus island (PI) 2 variants. A) Neighbor-joining phylogeny of the PI-2a BP gene, *gbs59*, based on an *in silico* analysis of 23 published sequences available in GenBank. Six major alleles were identified with 1,273 differences in 2,163 nucleotides and sorted into two groups: group 1 contains alleles, 1, 2, and 3, and group 2 contains alleles 4, 5, and 6. Bootstrap values based on 1000 replications are indicated at the nodes. **B)** Neighbor-joining phylogeny of thee alleles of the PI-2b BP gene, *san1519*, based on an *in silico* analysis of three published sequences. *san1519* alleles 1 and 2 differ at 199 of 4,317 nucleotides, whereas alleles 2 and 3 differ at 54 sites. Strain FSL S3-026, indicated in red, represents a bovine strain.

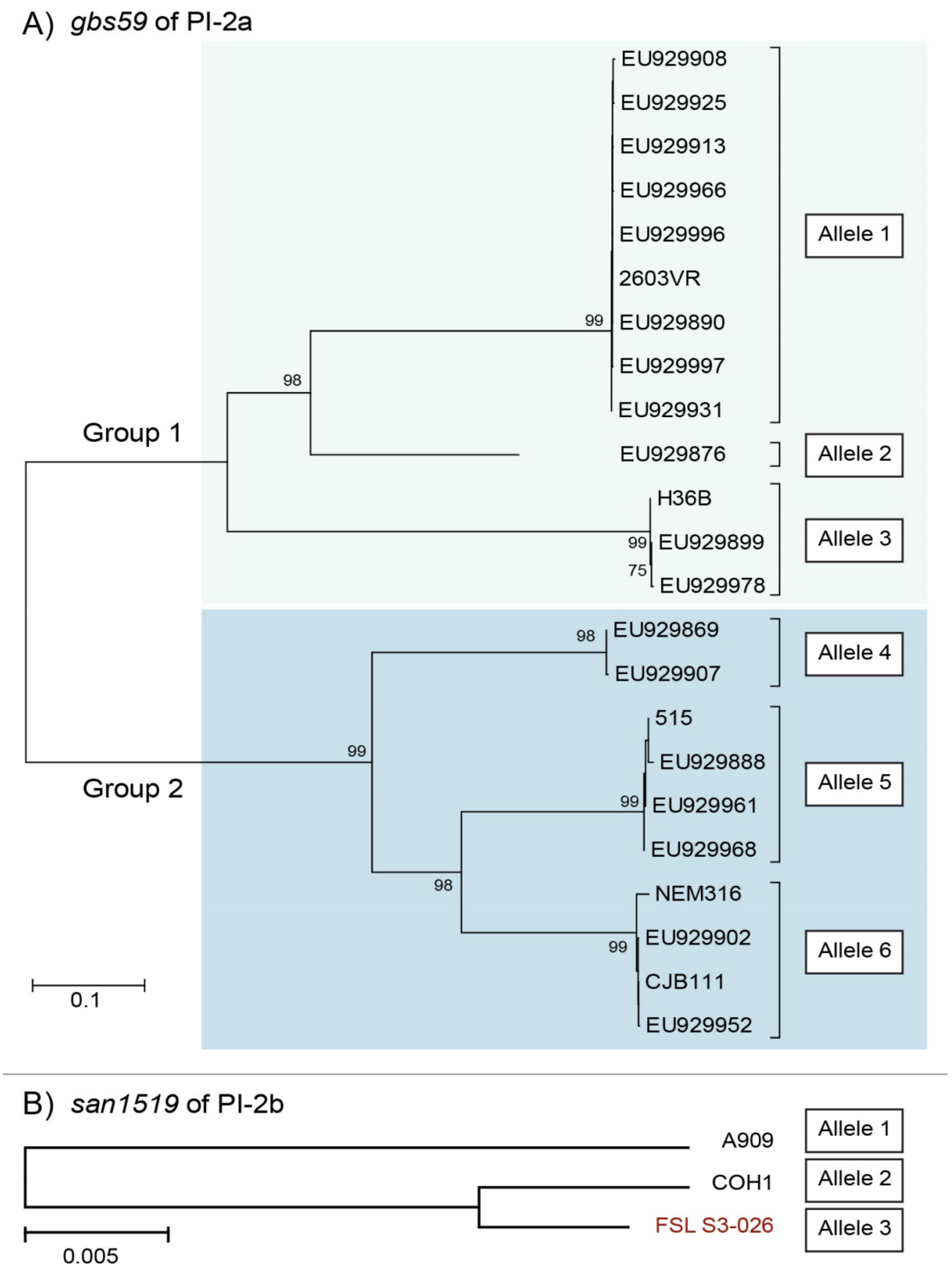

A) gbs59 of PI-2a
EU929908
EU929925
EU929913
EU929966
EU929996
2603VR
EU929890
EU929997
EU929931
EU929876
H36B
EU929899
EU929978
Allele 1
Allele 2
Allele 3
Group 1
98
99
99
75
EU929869
EU929907
515
EU929888
EU929961
EU929968
NEM316
EU929902
CJB111
EU929952
Allele 4
Allele 5
Allele 6
Group 2
98
99
99
98
99
0.1
B) san1519 of PI-2b
A909
COH1
FSL S3-026
Allele 1
Allele 2
Allele 3
0.005
